# Sensitivity enhancement in magnetic sensor using CoFeB/Y_3_Fe_5_O_12_ resonator

**DOI:** 10.1038/s41598-022-15317-0

**Published:** 2022-06-30

**Authors:** Md Shamim Sarker, Hiroyasu Yamahara, Lihao Yao, Siyi Tang, Zhiqiang Liao, Munetoshi Seki, Hitoshi Tabata

**Affiliations:** 1 Department of Electrical Engineering and Information Systems, Graduate School of Engineering, University of Tokyo, 7-3-1 Hongo, Bunkyo-ku, Tokyo, 113-8656 Japan; 2 Department of Electrical and Electronic Engineering, Khulna University of Engineering and Technology, Khulna-9203, Bangladesh

**Keywords:** Sensors and biosensors, Magnetic properties and materials

## Abstract

Magnonics, an emerging research field that uses the quanta of spin waves as data carriers, has a potential to dominate the post-CMOS era owing to its intrinsic property of ultra-low power operation. Spin waves can be manipulated by a wide range of parameters; thus, they are suitable for sensing applications in a wide range of physical fields. In this study, we designed a highly sensitive, simple structure, and ultra-low power magnetic sensor using a simple CoFeB/Y_3_Fe_5_O_12_ bilayer structure. We demonstrated that the CoFeB/Y_3_Fe_5_O_12_ bilayer structure can create a sharp rejection band in its spin-wave transmission spectra. The lowest point of this strong rejection band allows the detection of a small frequency shift owing to the external magnetic field variation. Experimental observations revealed that such a bilayer magnetic sensor exhibits 20 MHz frequency shifts upon the application of an external magnetic field of 0.5 mT. Considering the lowest full width half maximum, which is about 2 MHz, a sensitivity of 10^–2^ mT order can be experimentally achieved. Furthermore, the higher sensitivity in the order of 10^–6^ T (µT) has been demonstrated using the sharp edge of the rejection band of the CoFeB/Y_3_Fe_5_O_12_ bilayer device. A Y-shaped spin waves interference device with two input arms consisting of CoFeB/Y_3_Fe_5_O_12_ and Y_3_Fe_5_O_12_ has been theoretically investigated. We proposed that such a structure can demonstrate a magnetic sensitivity in the range of ${10}^{-9}$ T (nT) at room temperature. The sensitivity of the sensor can be further enhanced by tuning the width of the CoFeB metal stripe.

## Introduction

Spin waves (SWs), the collective excitation of electron spins in a magnetic material, are considered to be the most potential source of data carriers for future technology. SWs offer a wave-based ultra-low power data transmission capability and a wide range of frequency operations spanning from a few GHz to several tens of terahertz. SW is a wave, and so it offers data encoding capability in both amplitude and phase^1^. Moreover, the dispersion properties of SWs can be modulated by a wide range of parameters, including the magnetic field^2^, currents (electric^3^ and magnon^4^), laser light^5^, strain^6,7^, temperature^8^, voltage-controlled magnetic anisotropy^9^, and various micropatterning^10–12^, which make them suitable for a wide range of applications. The quanta of SWs are called magnons. The use of magnons in data processing applications is referred to as magnonics. Magnon-based computation devices, such as magnon FETs^13^, magnon logic gates (e.g., AND and OR^14^, NAND and XNOR^15^, NOR^16^, XNOR^4^, and majority gates^17,18^), frequency multiplexers^3,19^, half adders^20^, directional couplers^21^, have already been proposed. These devices are energetically more efficient than their electronic counterparts. Recently, magnonics has drawn significant attention for sensor applications, and magnon-based magnetic sensors^22^ and magnon-based gas sensors^23^ have been proposed. There are few available techniques to detect an extremely low magnetic field, such as the superconducting quantum interference device (SQUID) or magnetic resonance imaging (MRI). Both have an extremely high cost, making them inaccessible for most people. This is because SQUID requires a cryogenic environment, and MRI requires geomagnetic shielding. Spin-wave-based magnetic sensors work at room temperature and under low bias magnetic fields and are less expensive because of their smaller size (micron order) and simpler structure. Experimental evidence of achieving sub-millitesla magnetic sensitivity has been reported using the interference of two different types of spin waves in the magnonic cross-bar^22^. Two different types of spin waves (i.e., magnetostatic surface spin waves (MSSW) and backward volume magnetostatic spin waves (BVMSW)) propagating through two orthogonal arms makes the design and analysis of the structure quite complex. Moreover, working with a cross-bar SWs device requires an overlapping frequency region, which is very narrow due to the difference in SWs dispersion properties of MSSW and BVMSW. Therefore, a simple SWs transmission-based room temperature and high-sensitivity magnetic sensor is desirable. Recently, Matatagui et al. proposed a magnetic CuFe_2_O_4_ nanoparticle-based gas sensor using a nanoparticle/Y_3_Fe_5_O_12_ (YIG) heterostructure^23^. Different toxic volatile organic compounds (e.g., dimethylformamide, benzene, isopropanol, toluene, ethanol, and xylene) are absorbed by the CuFe_2_O_4_ nanoparticles, and the magnetic properties of the nanoparticles change due to the symmetry breaking of the nanocrystal structure at the surface, the surface strain, and the presence of dangling bonds. This change in magnetization causes a frequency shift of the propagating SWs in the YIG. A typical gas sensitivity is reported to be hundreds of ppm. In such a sensor, the shift of the full width half maximum (FWHM) or the peak position of the transmission spectra is used to detect the frequency shift. Transmission spectra with sharper peaks or lower FWHM values may result in enhanced sensitivity of the gas sensor. Some techniques have been reported to achieve a rejection band with low FWHM, which can be used to detect a small frequency shift owing to the tiny magnetization change. The most popular techniques are the use of periodic arrays of physical grooves^24^, width modulation^25^, or metal stripes^10^. The achievement of rejection bands in such magnonic structures is based on Bragg scattering. The wavelength ($\lambda$) of the SWs should match 2a/n (crystal period a, integer n). A large number of scattering units makes the device quite large. Scattering in the magnonic crystal also significantly suppresses the overall SWs intensity. Recently, Qin et al. proposed a technique to create a sharp magnonic bandgap using a single ferromagnetic metal stripe^26^. The driving mechanism mimics the optical Fabry–Perot resonator-like behavior in the magnonic domain. In this paper, we report the implementation of a magnonic analog to an optical Fabry–Perot resonator using a CoFeB/YIG bilayer structure to enhance the magnetic sensor sensitivity. The frequency shift of the sharp rejection band and the reduced FWHM of the notch were used as the response parameters. We mimicked the optical Fabry–Perot resonator behavior in the magnetic domain by creating two parallel reflecting interfaces using YIG and a patterned CoFeB bilayer structure. YIG serves as the SWs bus because of its extremely low damping constant. Placing the patterned CoFeB layer generates the reflecting interfaces. A few-atomic-layer-thick room-temperature sputter-grown TiO_x_ served as the spacer layer between YIG and CoFeB. One advantage of such a structure is that it eliminates the demand for many scattering units. Therefore, it paves the way for revolutionary device miniaturization. Moreover, the structure does not require a careful design of the wavelength and pitch of scattering units, and a simple placement of a ferromagnetic metal stripe between the coplanar waveguides (CPWs) is sufficient to implement such a magnetic sensor.

## Device fabrication

**Figure 1:**
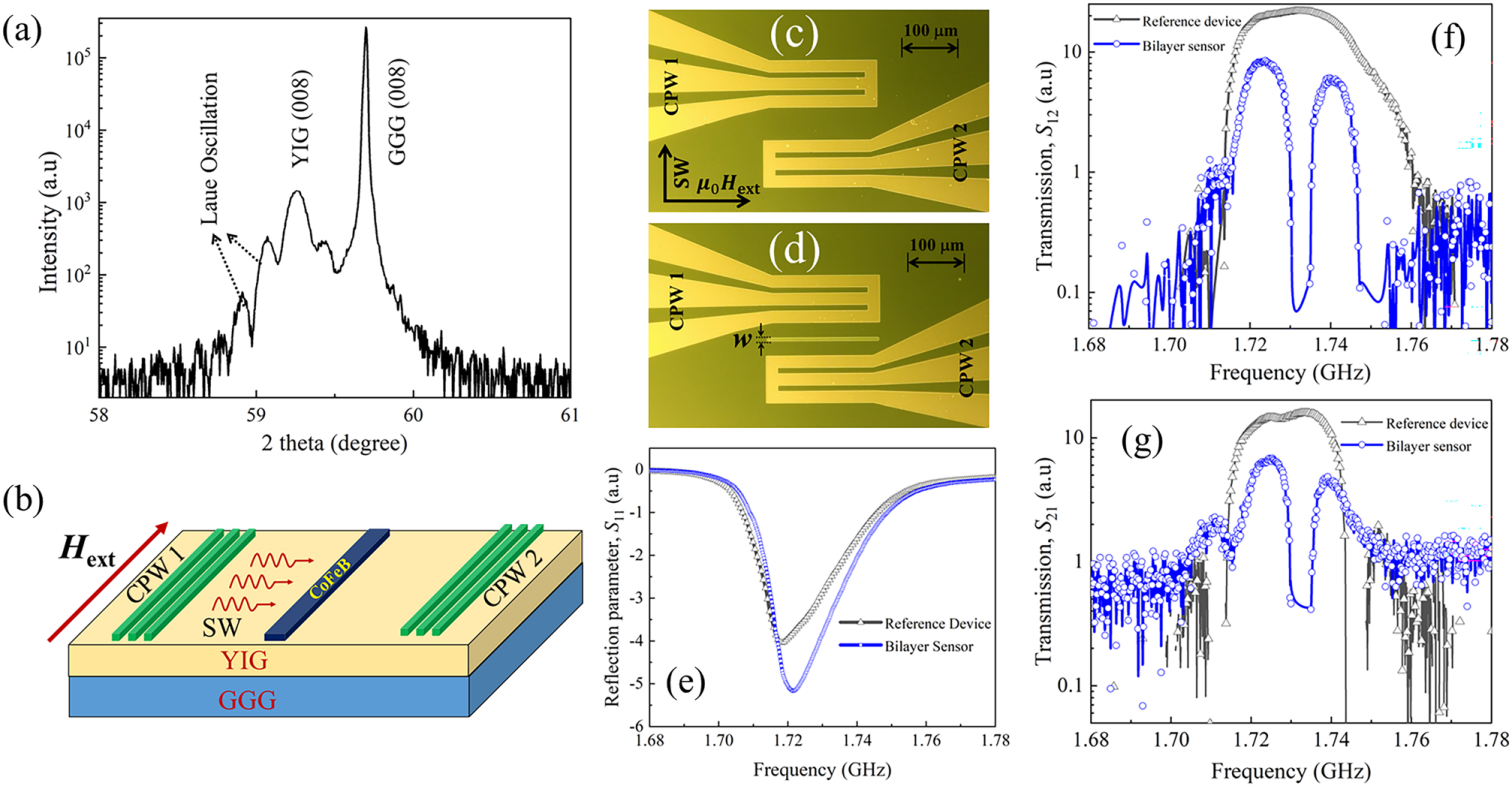
(**a**) $2\theta -\omega$ scan of epitaxial YIG film on the GGG [001] substrate. (**b**) Schematic diagram of the proposed CoFeB/YIG bilayer magnetic sensor. Optical image of the (**c**) reference and (**d**) CoFeB/YIG bilayer magnetic sensor. Scattering parameters (**e**) *S*_11_, (**f**) *S*_12_, and (**g**) *S*_21_ of the reference device and bilayer magnetic sensor. Here, the black curve with an open triangle represents the reference device, while the blue curve with an open circle represents the bilayer sensor. The bias magnetic field was maintained at μ_0_*H*_ext_ = 20 mT.

We grew a 70-nm-thick YIG thin film on a [001]-oriented single-crystalline Gd_3_Ga_5_O_12_ (GGG) substrate using the pulsed laser deposition (PLD) technique. The YIG film was deposited from a polycrystalline target at an oxygen partial pressure and substrate temperature of 0.1 Pa and 750 °C, respectively. An argon fluoride excimer laser at $\lambda$ = 193 nm was applied at 5 pulses per second. The sample was annealed in air at 800 °C for 3 h to enhance the crystallinity of the as-grown thin film. The deposition process resulted in a single crystalline YIG film, which was confirmed by X-ray diffraction using a PANalytical Empyrean diffractometer in $2\theta -\omega$ mode, as shown in Fig. 1a. The XRD scan shows a (008)-aligned peak from the YIG film and GGG substrate. The clearly visible Laue oscillation adjacent to the YIG film peak indicates a highly epitaxial film. Our single-crystalline YIG thin film has a lattice constant of 12.54 Å, which is significantly larger than the reported bulk lattice constant (12.38 Å). The non-stoichiometry of the Fe and O sites might be responsible for the lattice expansion. A ferromagnetic metal stripe of CoFeB with a thickness of 60 nm was patterned onto the YIG films by photolithography and room-temperature DC magnetron sputtering. Few atomic layers of TiO_x_ were used as the spacer layer between the YIG and CoFeB stripes. Subsequently, a system of two CPWs composed of 90-nm-thick Au was integrated into the YIG film using photolithography and DC magnetron sputtering. Approximately 4-nm-thick chromium (Cr) was used as the adhesion layer during the CPWs fabrication. The width of the signal and ground lines of the CPW was maintained at 20 μm. Their separation was maintained at 11 μm to match the antenna’s impedance with the vector network analyzer (VNA). The separation between the nearest edges of the signal lines of the exciting and detecting antenna was designed to be 120 μm.

## Results and discussion

First, we investigated the basic characteristics of the CoFeB/YIG bilayer device. For comparison, we fabricated a reference device consisting of a bare YIG film. We employed a microwave characterization technique using a VNA connected to two CPWs labeled CPW1 and CPW2. A DC-biased magnetic field (μ_0_*H*_ext_ = 20 mT) was applied in the plane of the film, but perpendicular to the direction of the SWs propagation. The SWs excited in this configuration are called the magnetostatic surface SWs (MSSW) or Damon–Eshbach (DE) transport configurations. The radiofrequency current applied from the VNA to the CPWs creates an oersted field around the signal wire, which excites the SWs in YIG by exerting a torque on the magnetic moments beneath the CPWs on the YIG surface. The SWs propagate by transferring the magnetic momentum to the nearest neighbor. The propagating SWs create a fluctuation in the local magnetization, causing a change in the magnetic flux and inducing a voltage in the detecting CPWs. To investigate the SWs propagation, we followed an approach similar to that in^27,28^, where the transmitted SWs spectra were obtained by recording the scattering parameters (e.g., reflection parameters *S*_11_, *S*_22_, and transmission parameters *S*_12_, *S*_21_). The plotted spectra were obtained by subtracting the background signal measured at μ_0_*H*_ext_ = 0 mT from the scattering parameters measured at the μ_0_*H*_ext_ = 20 mT bias magnetic field. The input power of the microwave signal from the VNA was − 15 dBm, which lies in the linear power region, allowing the nonlinear effect to be avoided^29^.

Figure 1b shows a schematic diagram of the CoFeB/YIG bilayer device. A stripe of CoFeB with a few layers of TiO_x_ spacer was placed on the YIG surface between the two CPW antennas. The width of the CoFeB microstrip was 15 µm. Optical images of the reference and bilayer devices are shown in Fig. 1c and d, respectively. The FMR spectra in terms of *S*_11_ parameters are displayed in Fig. 1e, where a μ_0_*H*_ext_ = 20 mT bias magnetic field was applied. A clearly visible notch centered around 1.72 GHz in the *S*_11_ spectra represents the ferromagnetic resonance. We calculated the Gilbert damping constant of the film from the *S*_11_ spectra as $= \Delta {f}_{\mathrm{FWHM}}/2{f}_{\mathrm{res}}=2\times {10}^{-3}$, where ${\Delta f}_{\mathrm{FWHM}}$ is the resonance linewidth, and ${f}_{res}$ is the resonance frequency of the *S*_11_ spectra. The obtained value of $\alpha$ is consistent with that of the tens-nm-thick YIG films grown by PLD^30^. Similar and symmetric shapes in the *S*_11_ spectra of both the reference and bilayer devices indicate that single-mode SWs should be excited in both cases. A minor shift in frequency in the *S*_11_ spectra of the reference and bilayer devices may occur because of the enhancement of the overall magnetization of the CoFeB/YIG bilayer owing to the positive magnetic susceptibility of CoFeB and the close proximity between CoFeB and YIG. Moreover, we calculated the shape-induced anisotropy of the microstructured CoFeB assuming a rectangular bar, as explained in^31,32^. The calculated anisotropic field along the long axis of the rectangular bar was found to be ${H}_{\mathrm{ani}}=-{N}_{\mathrm{y}}{M}_{\mathrm{sat}}=-2.45\times {10}^{-4}\times 180.5 \; \mathrm{mT}=-4.42\times {10}^{-2}$ mT, where ${N}_{\mathrm{y}}$ is the anisotropic factor, and ${M}_{\mathrm{sat}}$ is the saturation magnetization of CoFeB. The interaction of this shape anisotropy and the bias magnetic field might also be a contributing factor. However, we applied an equal magnetic field in the device but in the opposite direction (μ_0_*H*_ext_ =  − 20 mT) to determine the dominating effect. The data presented in Supplementary Material S1 indicate that the bilayer sensor device consistently exhibits reflection spectra (*S*_11_ and *S*_22_) at a frequency higher than the reference device, irrespective of the bias magnetic field direction. We conclude that the positive susceptibility-induced magnetization enhancement contributes to such a frequency shift, rather than the shape-induced anisotropic field of the CoFeB stripe.

The transmission spectra, *S*_12_ of the reference (black line with black triangle), and bilayer (blue line with open blue circle) devices are shown in Fig. 1f. The reference device shows an almost broadband transmission band starting from 1.71 to 1.76 GHz. However, a sharp dip in the transmission spectra at the frequency of 1.73 GHz was observed in the case of the bilayer device, unlike in the reference device. Similar transmission properties were also observed in the *S*_21_ parameter, as shown in Fig. 1g. The *S*_11_ parameters (Fig. 1e) do not show any spectral splitting, indicating that the notch or bandgap that appears in *S*_12_ and *S*_21_ does not originate from the excitation process. The frequency-specific feature of SWs propagation causes this bandgap. The bandgap formation can be explained in terms of the Fabry–Perot resonator mechanism described in the previous report, both numerically^33^ and experimentally^26^. The two edges of the CoFeB/YIG bilayer along the SWs propagation path form two parallel reflecting interfaces. Static dipolar coupling between CoFeB and YIG alters the effective magnetization of YIG, and the wavelength of the propagation of SWs is reduced. Wavelength down-conversion occurs when the incoming SWs exited in the YIG region enter the CoFeB/YIG bilayer cavity region. This down-converted SWs is reflected from the other edge of the CoFeB/YIG bilayer and then circulates inside this bilayer cavity and accumulate phase. If the circulating SWs accumulate a phase of $(2n+1)\pi$ upon two subsequent internal reflections within this bilayer, the incoming and circulating SWs interfere destructively and are suppressed from the propagation to the other side of the bilayer. In any other case of phase accumulation, constructive or partially constructive interference occurs, and SWs can exit the bilayer cavity and propagate on the other side of the bilayer. Thus, a bandgap frequency was formed in the transmission spectra of the bilayer device. A detailed mechanism has been explained in a previous report, which is consistent with our observations. A slight difference in amplitude and shape between the *S*_12_ and *S*_21_ spectra originates from the nonreciprocity of the surface SWs.

**Figure 2:**
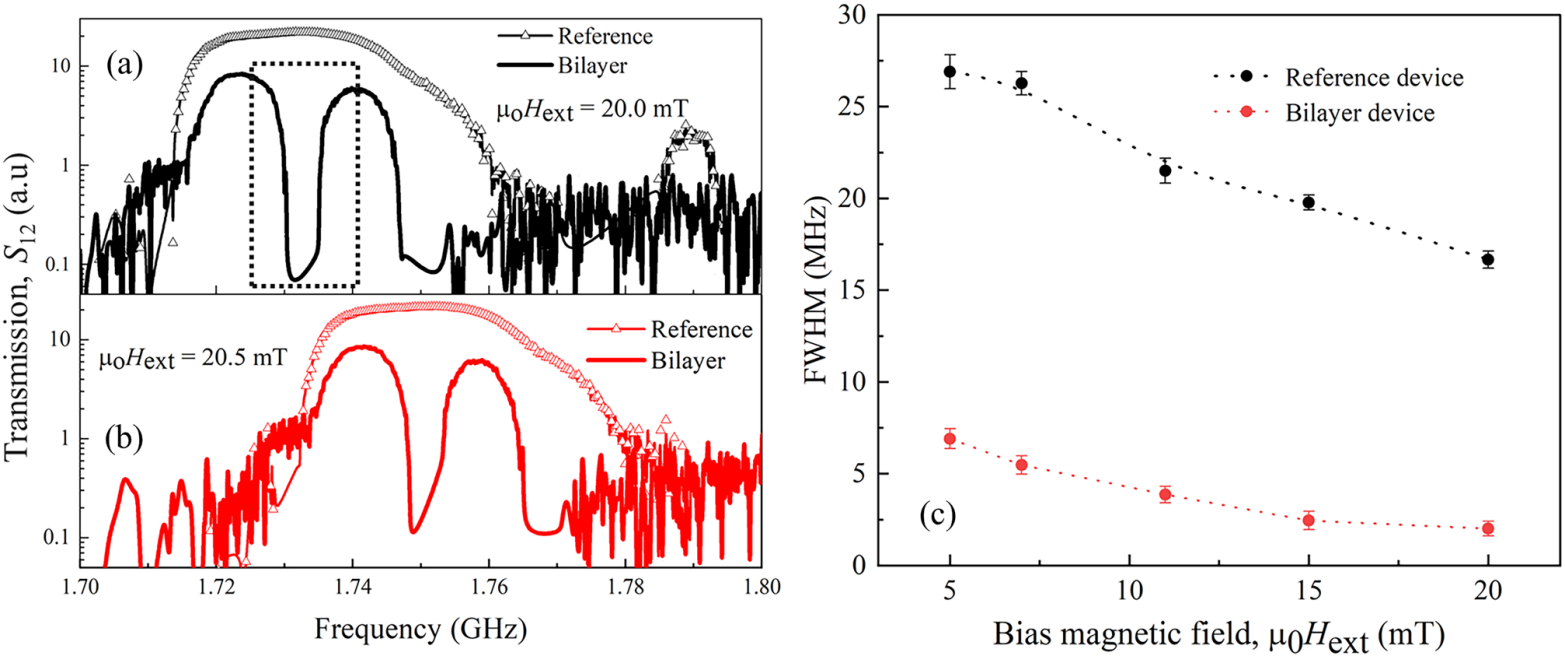
(**a**) *S*_12_ spectra of the reference device (black curve with open black triangle) and bilayer sensor (black curve) at μ_0_*H*_ext_ = 20 mT. (**b**) *S*_12_ spectra of the reference device (red curve with open red triangle circle) and bilayer sensor (red curve) at μ_0_*H*_ext_ = 20.5 mT. (**c**) The FWHM of the transmission spectra of the reference device (black dotted curve with filled circle) and the FWHM of the rejection band of the bilayer sensor (red dotted curve with filled circle). We calculated the FWHM and standard deviation ($\sigma )$ by Gaussian fitting. We considered $\pm 3\sigma$ as the error bar.

In the next step, we calculated the FWHM of the reference device and compared it with the FWHM of the bilayer device bandgap. We found that the FWHM of the bandgap of the bilayer device was 2 MHz, which is approximately 10 times smaller than the FWHM of the reference device transmission spectra. Such a sharp rejection band in the bilayer device can be used to design a highly sensitive magnetic sensor. To demonstrate the sensitivity enhancement of the magnetic sensor, we compared the transmission spectra of the reference and bilayer devices upon application of 0.5 mT additional external magnetic fields. The transmission spectra are shown in Figs. 2(a) and (b). The solid black line with an open black triangle in Fig. 2(a) represents the *S*_12_ spectra of the reference device, while the solid black line represents the bilayer sensor at μ_0_*H*_ext_ = 20 mT bias magnetic field. In contrast, the red counterparts in Fig. 2(b) represent the *S*_12_ spectra of the reference (red lines with open red triangles) and bilayer device (red line) with an additional 0.5 mT external magnetic field. Comparing the transmission with a 0.5 mT magnetic field difference in the reference device in Fig. 2a and b, we can observe an overlap frequency region. However, we cannot estimate the exact frequency shift owing to the additional magnetic fields because of the signal overlap. The approximation of the external magnetic field using the frequency shift of this flat transmission band and signal overlap results in a probability of a high error rate. However, a clear frequency separation can be observed if we compare the notch of the bilayer device bandgap. The bandgap notch moved from 1.731 to 1.751 GHz for the 0.5 mT external magnetic field application. The Kittel curve in this magnetic field range is almost linear (see Supplementary Material S2), and we can predict the external magnetic field change from the frequency shift. In our observation, the span of the frequency shift was 20 MHz for a 0.5 mT field change.

To verify our observation theoretically, we analyzed the frequency shift using the following Kittel equation:

1 $${f}_{\mathrm{res}}=\frac{\gamma }{2\pi }\sqrt{{H}_{\mathrm{ext}}({H}_{\mathrm{ext}}+4\pi {M}_{\mathrm{eff}})}$$

where ${f}_{\mathrm{res}}$ is the resonance frequency of SWs; $\frac{\gamma }{2\pi }$ = 28.08 GHz/T is the gyromagnetic ratio; ${H}_{\mathrm{ext}}$ is the bias magnetic field, and ${4\pi M}_{\mathrm{eff}}$ is the effective magnetization of YIG film. We have fabricated a similar YIG film using PLD on YAG substrate to measure the effective magnetization. We measured the hysteresis loop of the YIG/YAG film using the superconducting quantum interference measurement, which has been presented in Supplementary Material S3. From the hysteresis loop, we have extracted the effective magnetization of 170.5 mT, which lies in the range of the reported value^34,35^. Considering the ${4\pi M}_{\mathrm{eff}}$ to be 170.5 mT, we calculated the resonance frequency as 1.732 GHz for a 20 mT bias magnetic field. The 0.5 mT additional external field along with the bias field direction results in resonance frequency of 1.755 GHz, causing a frequency shift of 23 MHz, which is quite close to our experimental observation.

**Figure 3:**
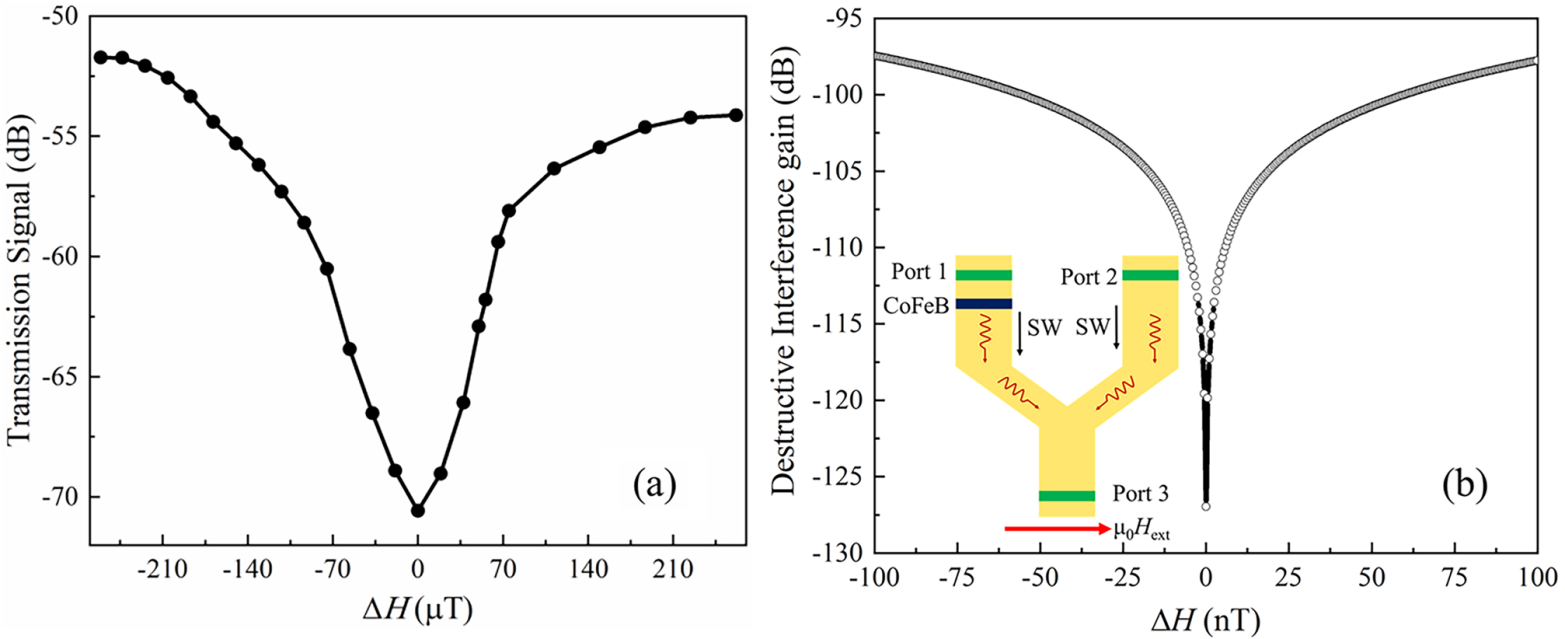
(**a**) Perturbation field ∆*H*-dependent transmission signal intensity. ∆*H* has been extracted from the sharp band gap of Fig. 2a, indicated by the black dotted rectangle in conjunction with the Kittel equation. (**b**) Theoretical calculation of interference gains at the destructive interference position vs. the nano Tesla sensing field of the proposed Y-shaped interference device shown in the (inset of the figure).

Figure 2c represents the magnetic-field-dependent FWHM of the transmission spectra of the reference device (black dotted curve with filled circle) and the FWHM of the bandgap of the bilayer device (red dotted curve with filled circle). Here, the FWHM and error bars (± 3σ, σ: standard deviation) are calculated by Gaussian fitting. Both the reference and sensor devices show a reduction in the FWHM as the bias field increases. The ratio of FWHM between the reference and sensor devices was calculated as 4 and 9 times at 5 and 20 mT, respectively. Thus, a higher bias field results in a sharper dip and better sensitivity. Another possible method to achieve better sensitivity is to consider the extraordinarily sharp edge of the frequency dip of the sensor device encompassed by the dotted black rectangle in Fig. 2a. We can achieve a significant difference in signal intensity for a slight change in frequency. We have converted the frequency change to perturbation magnetic field ∆*H* by using the Kittel equation, then the ∆*H* dependence of corresponding signal intensity has been plotted in Fig. 3a. This has been plotted in Fig. 3a. We considered ∆*H* = 0 T at the deepest position of the notch and calculated the shift in both directions of the frequency axis. The frequency and biased magnetic field can be tuned to the lowest intensity position (∆*H* = 0 T) by measurement setup, then we can detect a change in ∆*H* by recording the signal intensity. Figure 3a shows that 70 μT magnetic field change can result in about a 10 dB change in the signal intensity from ∆*H* = 0 T. We can achieve 1 (dB)/7 ﻿μT magnetic field sensitivity resolution in such a manner. However, the minimum dB resolution of our VNA is 0.01 dB which can be used to detect sub-﻿μT magnetic field change. Furthermore, we can achieve much higher sensitivity by combining the Y-shaped interference device with the concept of achieving a sharp edge in the frequency gap, as presented here. The Y-shaped interference device shown in the inset of Fig. 3b is made of micro-structured YIG. For instance, let us refer to the arm connected to Port 1 comprising the CoFeB/YIG bilayer as a bilayer bar and the other parallel arm comprising only YIG and connected to Port 2 as the reference bar. The phase of the signal coming from Port 1 is fixed, while that of the signal coming from Port 2 is tuned from 0 to 360°. Depending on the phase of each wave, they should show constructive and destructive interference. The SWs signals from Port 1 through the bilayer bar will provide a signal similar to that shown in Fig. 3a, whose intensity will respond to a tiny change in the magnetic field. However, the signal from Port 2 through the reference bar should exhibit a flat band spectrum as a reference device with an intensity that does not change with small magnetic field changes. We will adjust either the length or the microwave power of the reference bar so that its signal intensity is equal to the lowest signal intensity of Fig. 3a. This intensity is about − 70.58 ± 0.00001 dB, where 0.00001 dB is the uncertainty level of the signal intensity, which will not be changed with the tiny perturbation field ∆*H* due to its flat band nature. Initially, the bilayer will be operated at ∆*H* = 0 ﻿μT, where the signal intensity is a minimum at − 70.58 dB. In such a case, both the signals from Ports 1 and 2 will have almost the equal amplitude, and a strong interference will be observed (see Supplementary Material S4 for more details). However, the tiny perturbation field ∆*H* will increase the signal intensity of the bilayer bar. This will result in a difference in the signal intensity from the bilayer and reference bar, and this difference will make the interference weaker (see Supplementary Material S4). We only consider the intensity of the destructive interference as it shows the strongest shift in signal intensity upon the small ∆*H*. We summarized the intensity of the destructive interference position upon the perturbation field ∆*H* in Fig. 3b, which exhibits a magnetic sensitivity of 10^–9^ T (nT). The details of these proposed simulation studies have been shown in the supplementary material.

**Figure 4:**
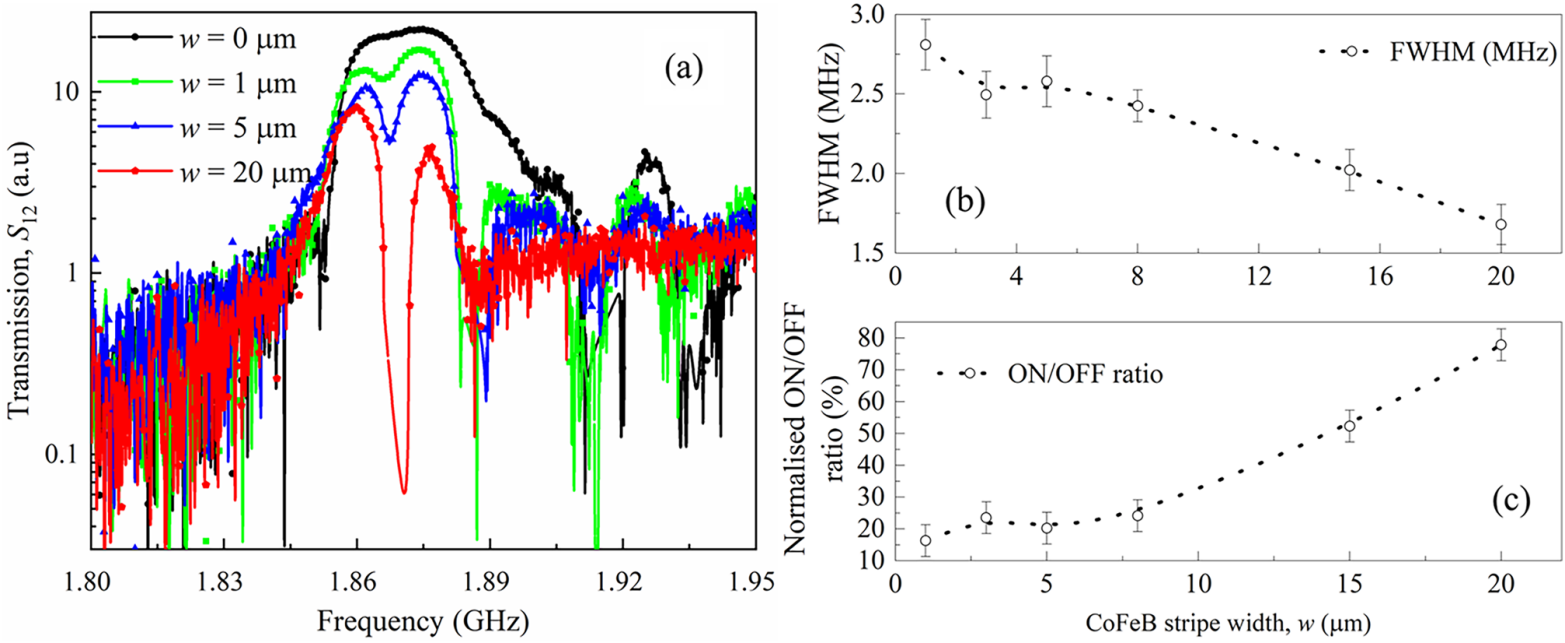
(**a**) Width-dependent *S*_12_ spectra of the bilayer sensor with the CoFeB width of *w* = 0, 1, 5, and 20 μm represented by the black, green, blue, and red curves, respectively. CoFeB width dependent (**b**) FWHM and (**c**) normalized ON/OFF ration of the transmission spectra.

In the subsequent step, we investigated the effect of the CoFeB stripe width on the performance of the bilayer magnetic sensor. We prepared CoFeB/YIG bilayer sensors with CoFeB stripe widths (*w*) of 1, 3, 5, 8, 15, and 20 μm, and then compared their transmission spectra. For simplicity, the transmission spectra of only 1, 5, and 20 μm width are shown with the reference device in Fig. 4(a). The bias magnetic field was maintained at μ_0_*H*_ext_ = 22 mT (the previous discussion was based on the 20 mT bias field and the 15 μm CoFeB stripe width). We can observe that the depth of the bandgap of the magnetic sensor increases and becomes sharper with increasing CoFeB width. The width of the signal wires of the CPWs was 20 μm; thus, the approximated wavelength of propagating SWs is 20 μm. Therefore, the rejection bandgap became sharper as the CoFeB stripe width increased toward the length of the SW wavelength. The FWHM of the magnetic sensor with respect to the CoFeB width is shown in Fig. 4b. Error margins were estimated in a similar manner, as shown in Fig. 2c. We can observe a gradual reduction in FWHM with the CoFeB stripe width, indicating enhanced magnetic sensitivity. Furthermore, we calculated the passband and stopband signal intensity ratios. In particular, we calculated the intensity difference between the highest signal intensity of the reference device and the lowest signal intensity of the magnetic sensor with a 20 μm CoFeB stripe width as ${\Delta I}_{max}$, which was considered to be 100. Thereafter, we calculated the normalized ratio of the signal intensity of the passband and stopband as

$$\mathrm{ON}/\mathrm{OFF \; ratio}=\frac{100\times {\Delta I}_{w}}{{\Delta I}_{max}}$$

where ${\Delta I}_{w}$ is the difference between the passband and stopband signal intensity of the magnetic sensor with a particular CoFeB stripe width, *w*. Figure 4c indicates that this normalized ON/OFF ratio increases with increasing CoFeB stripe width. A higher ON/OFF ratio indicates a better contrast between the passband and stopband signal intensity and hence a better sensing performance. Nevertheless, the overall signal intensity of the propagating SWs takes the edge off with increasing CoFeB stripe width as a consequence of signal absorption in the metallic CoFeB layer. The CoFeB stripe width-dependent SWs signal intensity is presented in Supplementary Material S5. S5 demonstrates that maximum intensity drops as low as − 53 dB at CoFeB stripe width of 20 µm. Further reduction in intensity will submerge the SWs signal inside the background noise and make it difficult to detect. Therefore, we must maintain a balance between high sensitivity and signal intensity. Furthermore, a subtle shift in the dip frequency has been observed with the increasing CoFeB stripe width; this may be associated with the resonance frequency shift due to the overall magnetization of the CoFeB/YIG bilayer.

Commercially available techniques to detect ultra-low magnetic fields, such as SQUID and MRI, have their own limitations. The performances of these devices are remarkable. However, SQUID requires a cryogenic environment with heavy-duty liquid helium pumping; thus, it is extremely expensive, power-consuming, and unrealistic for room-temperature applications. MRI techniques are bulky and require heavy mechanical movement and the necessity of geomagnetic shielding makes it power-demanding and impractical for daily use. Recently, tunneling magnetoresistance (TMR)-based sensors have been reported^36^. These require the sandwiching of multiple magnetic and spacer layers in atomic thickness, which is also challenging to achieve. Researchers are aiming for a simple and robust, highly sensitive magnetic sensor that can operate at room temperature. Therefore, spin-wave-based magnetic sensors have been proposed using magnetic crossbars^22^. Sub-millitesla magnetic field sensitivity has been demonstrated using that structure (although the theoretical prediction of the sensitivity is too high). Experimental demonstration. Our magnetic sensor based on the CoFeB/YIG bilayer is simple and can operate at room temperature and achieve a sensitivity of ﻿μT. We theoretically investigated the idea of combining the bilayer structure with the Y-shaped SWs interference device to achieve a sensitivity down to nT regime. Integrating this bilayer structure with a magnonic nanoparticle-based gas sensor^23^ may significantly enhance the gas sensitivity because a small shift in the dip frequency is accessible in the CoFeB/YIG bilayer structure. Moreover, the researchers may argue that a similar magnetic sensing performance might be possible in the case of groove-based or other Bragg scattering-based magnonic crystals. However, a groove-based design significantly deteriorates the crystal quality and reduces the signal intensity. Moreover, Bragg scattering requires a periodic structure of grooves or another metallic stripe with a period equal to or multiple to the SWs wavelength, making the device size very large. An accurate calculation of the wavelength, period and micropatterning complicates the structure. The CoFeB/YIG bilayer structure creates a similar but more efficient dip with a more straightforward and smaller structure. Therefore, it eliminates the need for accurate calculation of the micropatterning geometries.

## Conclusion

In this study, we experimentally demonstrated a highly sensitive magnetic sensor using a CoFeB/YIG bilayer structure. A sharp dip in the transmission spectra similar to magnonic crystals was created using simple CoFeB/YIG bilayers. The FWHM of this dip is about ten times smaller than that of the bare YIG device. We observed a 20 MHz shift in the dip frequency upon the application of an external magnetic field of 0.5 mT. Considering the lowest FWHM into account which is about 2 MHz, a sensitivity of 10^–5^ T order can be experimentally achieved. Furthermore, the higher sensitivity in the order of 10^–6^ T (μT) has been demonstrated using the sharp edge of the rejection band of the CoFeB/YIG bilayer device. In addition, a Y-shaped spin waves interference device comprising of a CoFeB/YIG and YIG in two arms was theoretically investigated. We propose that such a structure can demonstrate a magnetic sensitivity in the range of ${10}^{-9}$ T (nT) range. We also discussed the effect of the CoFeB stripe width on the performance of the bilayer sensor owing to the reduced FWHM. However, enlarging the CoFeB stripe width is associated with an additional loss in the passband, which may completely suppress the SWs propagation. Therefore, a careful balance should be maintained between sensor performance and transmission signal intensity. This device structure can also be used for microwave filtering applications. Tuning the property of the CoFeB by an external stimulus, such as current or voltage, might be interesting to study for the implementation of a reconfigurable magnonic resonator device. Moreover, the use of other ferromagnetic metals or Heusler compound as the bilayer can be investigated as the bilayer material with YIG to observe the behavior.

## Supplementary Information

Supplementary Information.

## Data Availability

The data that support the findings of this study are available from the corresponding author upon reasonable request.
