# Supplementary material for: Sensitivity enhancement in magnetic sensor using CoFeB/Y3Fe5O12 resonator
Source: Sci Rep. 2022 Jun 30;12:11105. doi: 10.1038/s41598-022-15317-0 (PMC9247025; doi:10.1038/s41598-022-15317-0)
Supplement: Supplementary file 1 — Supplementary Information. [file 41598_2022_15317_MOESM1_ESM.pdf]

Supplementary Information

Sensitivity enhancement in magnetic sensor using $CoFeB/Y_3Fe_5O_{12}$ resonator

Md Shamim Sarker[a,b], *Hiroyasu Yamahara*[a,*], *Lihao Yao*[a], *Siyi Tang*[a], *Zhiqiang Liao*[a], *Munetoshi Seki*[a], *and Hitoshi Tabata*[a]

[a]Department of Electrical Engineering and Information Systems, Graduate School of Engineering, University of Tokyo, *7-3-1 Hongo, Bunkyo-ku, Tokyo 113-8656, Japan*

[b]Department of Electrical and Electronic Engineering, Khulna University of Engineering and Technology, Khulna-9203, Bangladesh

S1: Reflection parameter S_{22} upon the application of positive and negative bias field.

The reflection parameter of the bilayer sensor device always stays ahead in frequency compared to the reference device, irrespective of the direction of the bias magnetic field. It indicates that the positive susceptibility of the ferromagnetic CoFeB assists the overall magnetization of the YIG and enhances the resonance frequency of the bilayer sensor device.

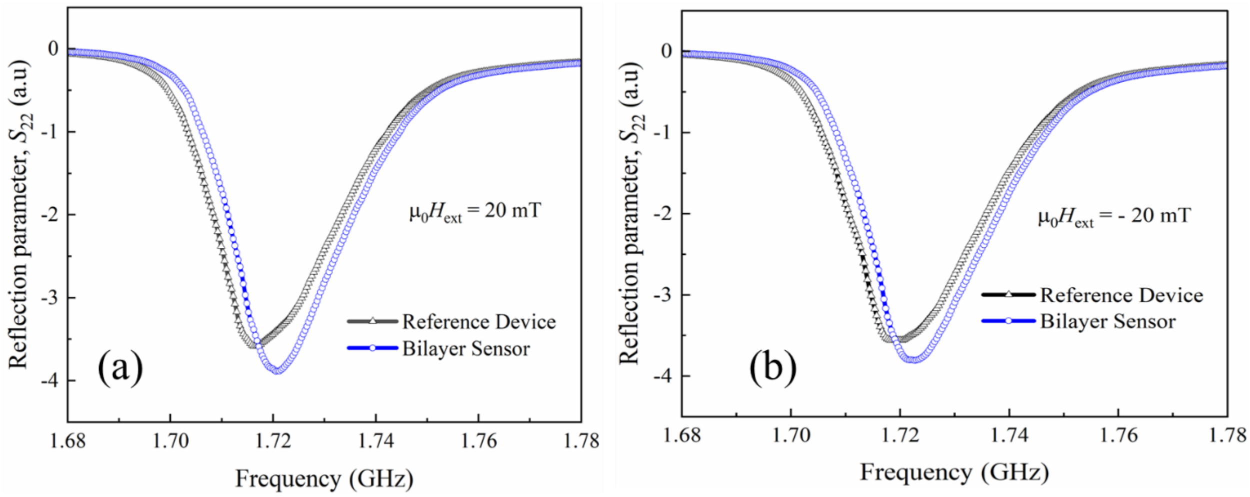

Supplementary Figure 1. Reflection parameter S_{22} upon the application (a) $\mu_0 H_{ext}$ = 20 mT and (b) $\mu_0 H_{ext} = -20$ mT bias magnetic field.

S2: Magnetic field-dependent resonance frequency

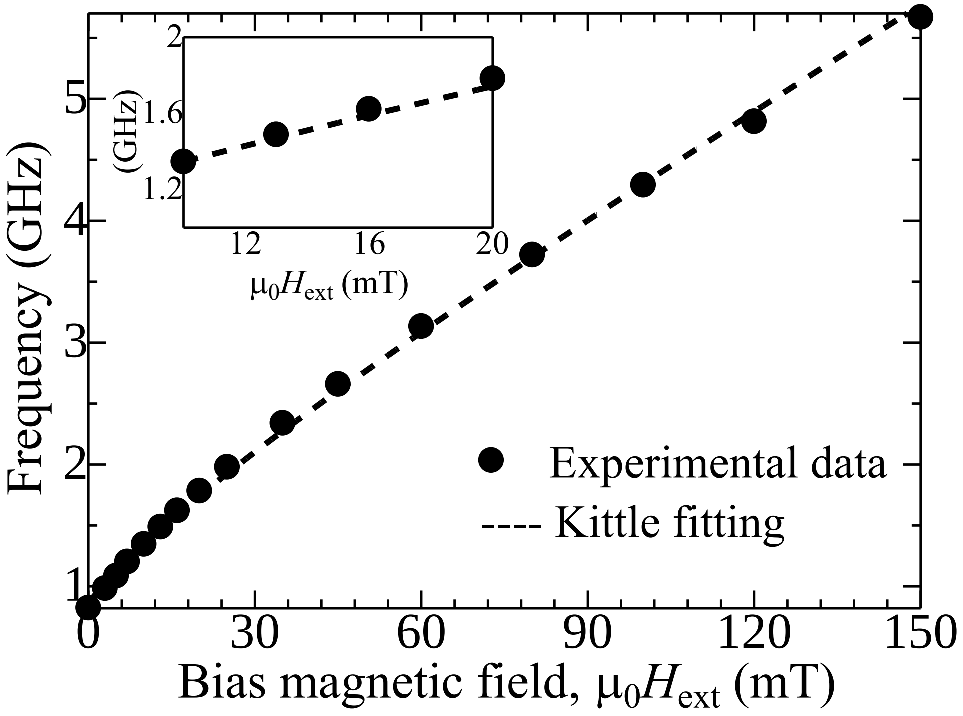

Supplementary Figure 2. Bias magnetic field dependent resonance frequency. Black filled circles represent the experimental data, while the dotted curve indicates the Kittle fitting. Inset that indicates the bias field vs. resonance field is almost linear in the small range of the bias field.

S3: Hysteresis loop of the YIG film

For the measurement of the hysteresis loop, we fabricated a YIG film over the YAG substrate. The superconducting quantum interference device (SQUID) was used to measure the data. From the hysteresis loop, we have extracted the effective magnetization of the YIG film. Our measured magnetization was 170.5 mT.

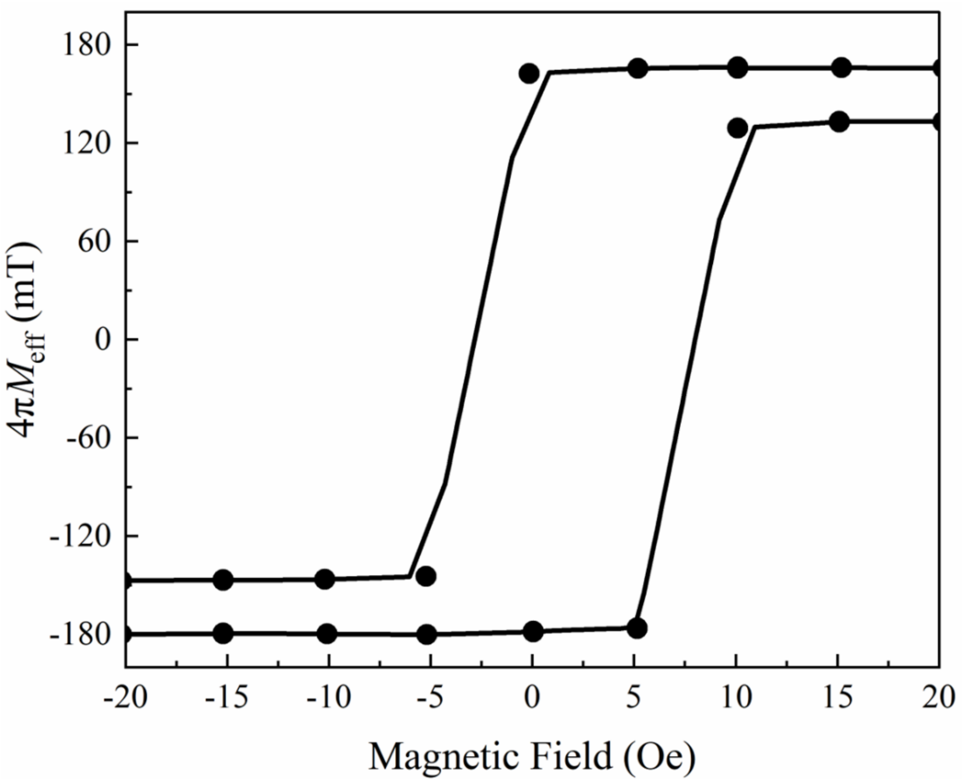

Supplementary Figure 3. Hysteresis loop of grown YIG. Measurement has been performed using the superconducting quantum interference device (SQUID) at room temperature.

S4: nano-Tesla sensitivity prediction using a hybrid Y-shaped interference device

In the proposed device, we mainly consider the interfering behaviors of two spin waves in the Y-shaped interference device shown in supplementary Figure 4(a). The magnetostatic surface wave (MSSW) was excited at Ports 1 and 2. Both two waves have in-plane magnetization, and their interference behaviors are similar to that of two plane waves. The two waves excited at Ports 1 and 2 are depicted by the equations $M_{\delta 1} = A_1 exp[i(\boldsymbol{k_1 r} + \omega_1 t + \varphi_1)]$ and $M_{\delta 2} = A_2 exp[i(\boldsymbol{k_2 r} + \omega_2 t + \varphi_2)]$, respectively. $M_{\delta 1}$ wave generated at Port 1 passes through the bilayer arm, while the $M_{\delta 2}$ wave generated in Port 2 passes through the reference arm. Variable δ represents the component of magnetization on a specific direction (x, y, z). The parameters $A, \boldsymbol{k}, w, \varphi$ are amplitude, wave vector, angular frequency, and phase. Variables $\boldsymbol{r}$ and t are the position and time, respectively. Damping constant is not considered as we aim to focus on the interfering behaviors, although it may further reduce the value of interference gain (dB) in real situations. The calculation process is fulfilled by MATLAB.

Interference occurs between waves with the same wave vectors and the same angular frequencies. Thus, we assume: $|\boldsymbol{k_1}| = |\boldsymbol{k_2}|$, and $\omega_1 = \omega_2$. Destructive interference occurs when the phase difference between φ_1 and φ_2 equals to 180°. If the amplitude of the two signals A_1 and A_2 are exactly the same, the interference gain ($|M_{\delta 1} + M_{\delta 2}|$) will be theoretically infinitesimal at the destructive point. However, if A_2 changes slightly because of the magnetic field change, the interference gain at the destructive interference point will intensively change according to the basic principles of interference. In calculation, A_2 is set to be -70.58001 dB (due to its flat band spectra as reference device discussed in the manuscript). We can get a flat band at -70.58001 dB from the reference arm by making the arm longer so that the signal is significantly damped during the propagation, or we can control the microwave power. Meanwhile, A_1 is set by a function of the

magnetic field change shown in supplementary Figure 4(b). The function is experimentally acquired, with a fitting process to upsample the values in areas where the magnetic field change is smaller than 18.7μT. We assume that A_1 is −70.58 dB when the magnetic field change is 0. It is known that the interference gain (dB) varies with the phase difference $\Delta\varphi = |\varphi_1 - \varphi_2|$. The interference pattern is shown in supplementary Figure 4(c) which corresponds a field change of 0 nT. Now, A_1 will always increase when the magnetic field change increases in either direction (+, -), according to our experiment results. Nevertheless, we assume that A_2 remains the same under any circumstances. A typical interference pattern of A1 = −70.57171 dB and A2 = −70.58001 dB (corresponding to 100 nT change in field) has been shown in supplementary Figure 4(d). We can observe the intensity of the destructive interference position has been significantly changed from -127 dB at 0 nT to -97.76 dB at 100 nT. We also calculate the destructive interference gain value (dB) that varies with the magnetic field change (in 0.5nT intervals, from -18.7μT to 18.7μT) shown in supplementary Figure 4(e). The expanded view in the nT regime shown in supplementary Figure 4(f) reveals that it is possible to decide the magnetic field change in the nT scale by detecting destructive interference gain of the two waves.

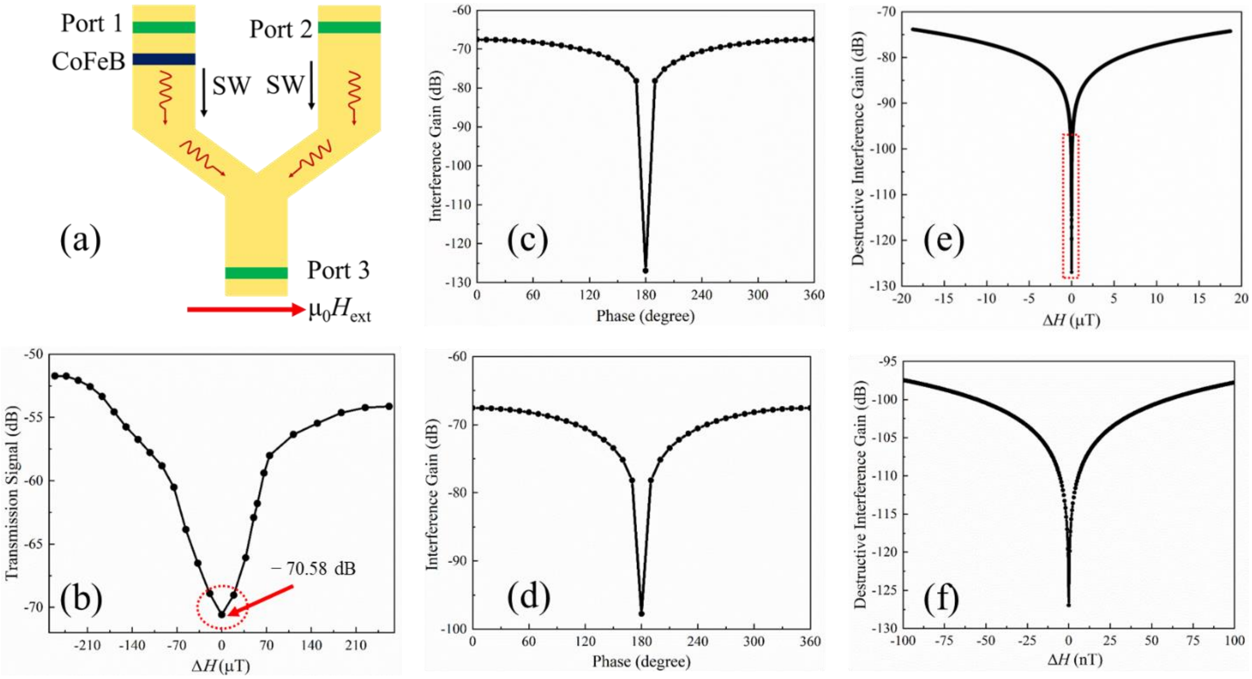

Supplementary Figure 4. (a) Proposed hybrid Y-shaped SWs interference device consisting of a CoFeB/YIG bilayer in one arm and reference YIG in another arm. (b) Sensing field ΔH−dependent transmission signal intensity. ΔH has been extracted from the sharp band gap of Figure 2(a) indicated by a black dotted rectangle in conjunction with the Kittle equation. This response is expected from the bilayer arm, while the reference arm should exhibit the flat band spectra. The interference pattern of Port 1 and Port 2 waves with (c) $\Delta H = 0$ nT and (d) $\Delta H = 100$ nT. Destructive interference gain value (dB) with respect to the magnetic field change (e) from -18.7μT to 18.7μT (f) from -100 nT to 100 nT

S5: CoFeB stripe width-dependent maximum signal intensity of the bilayer device

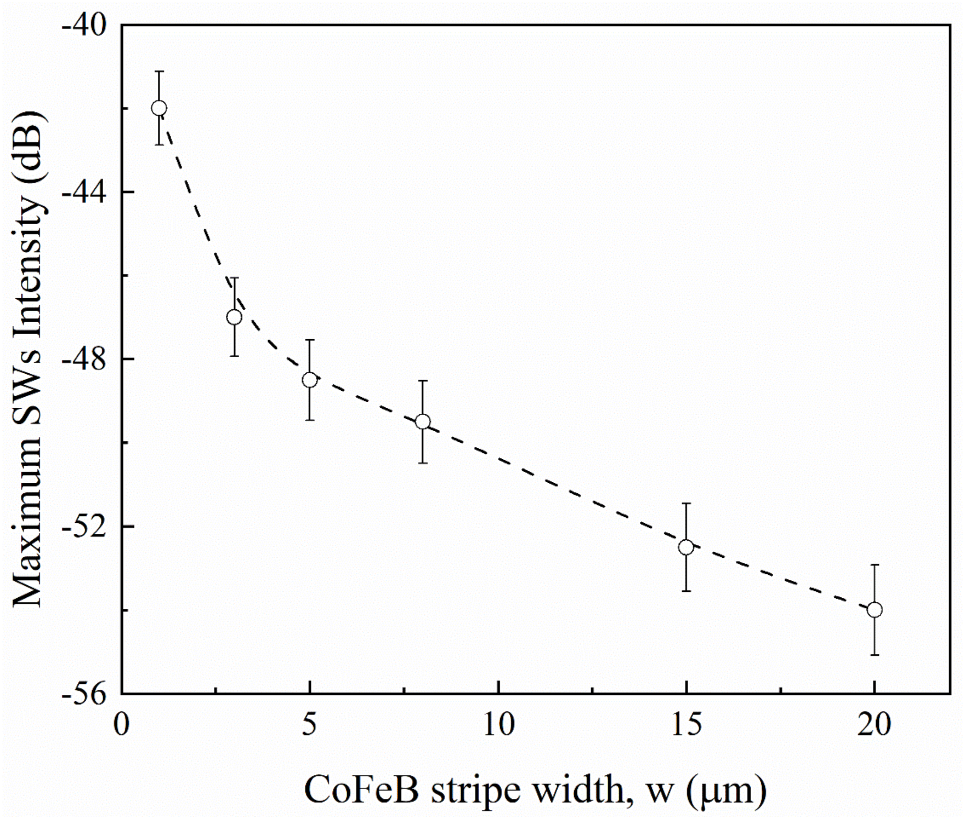

Supplementary Figure 5. CoFeB stripe width-dependent maximum signal intensity of the bilayer device.
